# How to Attract Suitable Women to the Nursing Profession

**Published:** 1920-07-03

**Authors:** 


### ·COLLEGE OF NURSING CONFERENCES.

### JUNE 17 and 18, 1920.

### How to Attract Suitable Women to the Nursing Profession.

**The Chairman (Dr. J. Kay Jamieson,** Dean of the Medical Faculty, Leeds University), who, during the war, was in charge of a large military hospital, said he had watched the development of the organisation of the profession since the College was formed and its activities increased, and it seemed to him that the College was destined to perform the same function for the nurses as the General Medical Council did for the medical profession. It was of the utmost importance that the nursing profession should have a good mouthpiece, and if the College was to do anything for the profession it must have behind it the complete loyalty and adhesion of all nurses. And there should be constant communication between nurses and their organisation, because it could never be known what would be useful or act as a stimulus. There must be for nurses a normal average effective training, and he considered it important that some means should be found of filling up the gap between the girl leaving school and commencing her training as a nurse. He had received a letter from Miss Walker, in which she said that many now fell back upon nursing as a profession after an unsuccessful training for other callings, and she suggested the establishment of colleges to which girls could pass from the ordinary schools to prepare for their hospital duties, hospital authorities choosing their probationers from among those who had successfully passed their hospital tests. He thought the scientific part of the curriculum could easily be overdone by being allowed to take up too large a proportion of the time; the essential and important part of the professional work could only be done in hospital. The short courses of lectures to nurses should not be regarded as of small importance by the professor, and therefore relegated to a junior member of the staff. He was in the habit of giving the nurses' lectures on anatomy at Leeds University himself.

### Health Visitors Should be Trained Nurses.

The scope of the nursing profession had so far been too narrow, and in his view the recent tendency had been towards making the outlook even narrower. This must be combated, especially in view of the added powers given to the Ministry of Health. When questions concerning health visitors arose, the nurse was the proper person to consider; she was the one person of all others in the country who had the ear of the people. Every health visitor should be a trained nurse, with, as an additional equipment, some special education in social science. This would be a field for the trained nurse which would probably attract more recruits, and good ones. The health visitor could not be made in a hurry; she needed several years of training, the basis of which was the ordinary training of the nurse. Then in ten years there would perhaps be a more effective health-visiting service in the country.

### Trade Unionism Not Suitable for Nurses.

The usual way of getting proper remuneration at the present time was by trade unions; but while a certain part of remuneration took the form of money and kind, there was also another recognition in the form of respect and affection on the part of the populace. The nursing profession had bought its status at a very heavy price, but it now stood first in the affection of the people. The way to secure progressive betterment was through such a body as the College of Nursing. The constitution and methods of trade unionism he did not consider suitable for the professions; those methods could not be indulged in by nurses without losing a great deal of that which made life worth living.

**Miss J. Lane-Claypon,** M.D., D.Sc. (Dean, Household and Domestic Science Department, King's College for Women), said that much of what she intended to say was applicable to any profession, though certain aspects of education were peculiar to the nursing profession. The entrance of women into professions was a quite recent event; therefore it was instructive to study what men had evolved for themselves in the course of experience. They had laid down a minimal standard to which all intending entrants to the particular profession must attain. The time was now fully ripe for nurses to determine their standard in the same way. The standard aimed at must lie in three ways—a standard portal of entry, a standard of training, a standard of conditions or of outlet for those whose training had been satisfactory. These must all be considered simultaneously, as they were interdependent.

### The Standard of Entry.

To cope with the difficulties of crowded careers there must be a fairly high standard, one fully as high as could be reached by the average person. Curiously, when something was made difficult of attainment, that very fact seemed to be an inducement for people to strive after it; conversely, if it were made easy it fell into disrepute. That was one of the saving graces which Britons, at all events, still possessed. The people who first entered a profession—for instance, the women who first entered the medical profession —were of outstanding ability; they had to do the hard pioneer work, to open the door and make the entry easy for those who would come after them. At King's College for Women they had no particular entrance examination four years ago, but it had been increasingly stiffened since, and now the full London University standard of examinations had been attained there. Concurrently with that stiffening, the number of entrants had increased. She did not know anything which could reasonably take the place of the examination; it seemed to be the only means of ascertaining the candidate's intelligence. She thought every candidate should have been educated up to her eighteenth year; under that age a person was not possessed of full intelligence nor had yet learned how to work and study.

### Unification in Hospital Training.

The actual training of the nurse she did not propose to say much about, as that was known to her audience; but with regard to outlet, the varying standards of hospital training should be unified as far as they could be, for that had much to do with the subsequent outlook. Last July the Board of Education issued special regulations with regard to the training of health visitors, which would bring the training of the nurse into line with that of the health visitor; and this was followed by a Memorandum from the Ministry of Health to all local authorities, instructing them that after a period—to be named—no local authorities would receive grants-in-aid unless their nurses had this specialised training which had been laid down by the Board of Education. These regulations provided that the woman who was already a trained nurse should be only required to put in one year at specialised training. This extra year was for work which did not come into the nurse's ordinary training, for neither the medical nor the nursing profession had yet realised the enormous importance of preventive medicine. There was a lamentable deficiency in the study of the normal, even of the normal baby. The curriculum could be so arranged that the training as both nurse and health visitor could be got into four years.

### Paucity of "Plums" and Pensions.

There were two or three things which were militating against the entry of women into the nursing profession. One was that they believed there were not enough "plums" or good appointments open to them to stimulate their efforts. A second was the uncertainty of provision for old age. It

**College of Nursing Conference**—(*continued*).

was very gratifying to know that the College was trying to overcome this disability. A further objection was on the score of the long hours of work; the nursing profession was too arduous for many to feel justified in facing it. Many people, especially those responsible for private nursing, certainly exploited their nurses to an undue degree. Here again she was very pleased to know of the attempts to curtail and regulate the hours, in both hospital and private work. She had no fears for the future of the nursing profession; she believed it would become one great, big, united, well-trained and satisfactory profession—satisfactory to the nurse herself and to the public alike.

**Miss Musson** (Matron, General Hospital, Birmingham) thought it was time there was a standard of admission to the nursing profession, but she did not think a preliminary examination could be asked for at present, owing to the shortage of probationers. A better standard of pay was the first need if more were to be attracted to the work. Pressure should be brought on the Government Departments to ensure that only fully-trained nurses should be engaged for all nursing posts under the Government; also that when, owing to a deficient supply of fully-trained women, partially-trained nurses had to be employed, the former should have a higher rate of pay. Trained nurses should not be expected to work under untrained superintendents. The conditions in the training-schools must be improved; the probationer must have shorter hours of work, so that she came to the lectures less fatigued than was inevitable at present. Moreover, the teaching must be better and more systematic; this was being gradually effected by the appointment, in many hospitals, of special teaching sisters; but it must not be forgotten that the real trainers were the ward sisters. There should not only be more leisure for the nurse, but more opportunities provided for her recreation. She suggested the giving of lectures on the history of nursing to girls in schools. The right type of girl should be shown what the profession had to offer her, and be convinced that the nation needed her services. It would be well for nurses to talk more of the good side of the profession, and not be too much obsessed by the difficulties and drawbacks.

**Miss Edmondson** (Matron, Royal Infirmary, Aberdeen) said she was fully in agreement with what Dr. Lane-Claypon said on the subject of previous training for the nurse; to have such a standard would not keep candidates away, but would lead to applications from a better class of young woman. All the kinds of nursing—children, fever, mental, and midwifery—should be embraced in the one training. All the housmaid's work should be cut out of the nurse's training; in Aberdeen that reform had been brought about, as well as securing to all nurses one day's rest in seven. Nurses should be given all possible recreation; at the nurses' homes there should be a good tennis court and a good billiard table, and nurses should be allowed to bring their friends, who need not always be female friends. (Laughter.) The probationers should not be paid much in their first and second years; let them value the training they were receiving, but pay them well when in their third year and they were more useful, but especially let the fully-trained nurse be adequately remunerated.

**Miss Sparshott** (Manchester) pointed out that at many hospitals the nurses already had facilities for recreation. The girl whose education had been sound and good was the one who would be willing to turn her hand to anything.

